# NR2B Antagonist CP-101,606 Abolishes Pitch-Mediated Deviance Detection in Awake Rats

**DOI:** 10.3389/fpsyt.2014.00096

**Published:** 2014-08-05

**Authors:** Digavalli V. Sivarao, Ping Chen, Yili Yang, Yu-Wen Li, Rick Pieschl, Michael K. Ahlijanian

**Affiliations:** ^1^Exploratory Biology and Genomics, Bristol Myers Squibb Company, Wallingford, CT, USA

**Keywords:** mismatch negativity, auditory deviance, NMDA antagonist, ketamine, NR2B, CP-101,606

## Abstract

Schizophrenia patients exhibit a decreased ability to detect change in their auditory environment as measured by auditory event-related potentials (ERP) such as mismatch negativity. This deficit has been linked to abnormal NMDA neurotransmission since, among other observations, non-selective channel blockers of NMDA reliably diminish automatic deviance detection in human subjects as well as in animal models. Recent molecular and functional evidence links NR2B receptor subtype to aberrant NMDA transmission in schizophrenia. However, it is unknown if NR2B receptors participate in pre-attentive deviance detection. We recorded ERP from the vertex of freely behaving rats in response to frequency mismatch protocols. We saw a robust increase in N1 response to deviants compared to standard as well as control stimuli indicating true deviance detection. Moreover, the increased negativity was highly sensitive to deviant probability. Next, we tested the effect of a non-selective NMDA channel blocker (ketamine, 30 mg/kg) and a highly selective NR2B antagonist, CP-101,606 (10 or 30 mg/kg) on deviance detection. Ketamine attenuated deviance mainly by increasing the amplitude of the standard ERP. Amplitude and/or latency of several ERP components were also markedly affected. In contrast, CP-101,606 robustly and dose-dependently inhibited the deviant’s N1 amplitude, and as a consequence, completely abolished deviance detection. No other ERPs or components were affected. Thus, we report first evidence that NR2B receptors robustly participate in processes of automatic deviance detection in a rodent model. Lastly, our model demonstrates a path forward to test specific pharmacological hypotheses using translational endpoints relevant to aberrant sensory processing in schizophrenia.

## Introduction

While recording event-related potentials (ERPs), when a stream of identical stimuli are interrupted occasionally by an atypical stimulus, the so-called odd ball, an enhanced negativity to the odd ball or deviant is observed from fronto-central regions of the scalp-recorded clinical electroencephalogram. This response is called the mismatch negativity (MMN) and has been recognized as a pre-attentive neurocognitive mechanism for change detection in the environment (1–3). Not surprisingly, an ability to rapidly detect change is evolutionarily conserved and can be detected across a variety of species in addition to primates (4–7). Key generators for auditory mismatch-related negativity have been found within the primary and secondary auditory cortex with additional sources in frontal and parietal cortical regions (8–13). Although MMN is perhaps the best characterized pre-attentive mechanism for deviance detection that manifests about 100–200 ms after stimulus presentation, it is not the only mechanism for automatic change detection (1, 14). It is now known that there is neural activity in anatomical structures along the sensory processing hierarchy involved in change detection, some apparent as early as 30 ms after stimulus presentation (15).

Mismatch negativity has been shown to be disrupted in a variety of mental disorders including schizophrenia, autism, and dementias (16, 17) and constitutes a convenient measure to evaluate neurophysiological function especially because it does not need engagement from the subjects in terms of participation or motivation to complete the test – an obvious advantage when studying psychiatric or neurologically challenged subjects. These characteristics also make it an attractive model for translation into preclinical species where apart from studying the neurophysiological mechanisms and neural circuits more intensively, therapeutic hypotheses can be tested to develop potential new remedies (6, 7, 18).

While mismatch-like deviance detection has been demonstrated in multiple species, such data from the most commonly used laboratory species, i.e., rodents has been controversial and conflicting. In general, when deviance detection was investigated at the single-unit level or at the local field potential within the primary auditory cortex, the results have not been encouraging in that a clear electrophysiological substrate for deviance could not be established (19, 20). On the other hand, epidural macroelectrode recordings have generally found evidence for true deviance (4, 21–23), suggesting that the neural sources may be distributed more widely (7). Previously, robust deviance detection was shown in anesthetized Guinea pigs from vertex ERPs but not from the temporal cortex, which is the location for primary cortical processing (24, 25). These authors concluded that there are parallel auditory pathways, and in Guinea pig, the temporal lemniscal stream was not contributing to deviance detection. Rather, the midline structures (with input from caudomedial thalamic nucleus) projecting to multisensory cortical regions below vertex were involved. However, it is not known if the vertex potentials in rats, which represent a non-lemniscal processing stream (26, 27), also model deviance detection.

NMDA receptors have been implicated in the generation of mismatch negativity. Local application of NMDA antagonists like PCP disrupts MMN in monkey auditory cortex in a layer-specific manner (28). Several clinical reports indicate a disruption of MMN in the presence of a non-selective antagonist like ketamine (29–33). Indeed, MMN has been argued as an index of NMDA receptor dysfunction in schizophrenia in part based on these findings (28, 32). However, NMDA receptors are heterotetramers made of two NR1 and two NR2 subunits with considerable heterogeneity in expression and distribution within the brain (34, 35). Non-selective cation channel blockers disrupt all NMDA neurotransmission irrespective of subunit composition. Thus, as pharmacological tools, they have limited utility and cannot decipher the relative contribution of specific subunits to deviance detection. While selective ligands are not available for all of the extant NMDA subunits, highly selective NR2B ligands have been available for sometime making it possible to test their role in deviance detection (36, 37). Moreover, although the contribution of NR2B receptor to deviance detection has not been investigated, several recent reports have indicated robust participation of NR2B receptors in mediating higher cognitive functions such as behavioral flexibility in rodents (38–40) and working memory in primates (41), using tasks that engage medial prefrontal cortex. Interestingly, medial prefrontal cortex has been implicated in auditory deviance detection (42, 43) and MMN deficits in schizophrenia patients are explained in the context of selective decrease in gray matter volume in prefrontal and temporal regions of the brain and their aberrant mutual connectivity (44–46). In addition to deviance detection, distinct cortical ERP components such as the P1–N1–P2 complex represent cortical registration of auditory sensory input (47). These components, especially the N1 and P2, unlike the early evoked potentials such as the brain-stem responses, can be modulated by endogenous factors such as attention (48, 49) and are often also reported to be aberrant in schizophrenia patients (50–52). However, there is incomplete understanding of how such observations are modeled by NMDA antagonists. Rodent ERP components like N1, although not identical to their human namesake in every respect, nevertheless share important features, are frequently modulated in similar ways and are regarded as homologs (53, 54). For example, non-selective NMDA channel blockers such as ketamine have been shown to modulate the N1 response in healthy controls (33, 55) as well as in rodents (56, 57). However, there are no published data that examine NR2B effects on these ERP components.

In the following report, we first tested if there was reliable and true deviance detection to simple auditory tones in the vertex potentials of conscious rats. We then tested a single odd-ball protocol and characterized the effects of two different NMDA channel modulators at multiple time points after dosing. Additionally, we also determined the effect of these drugs on three individual ERP components: P1, N1, and P2, elicited in response to frequent and infrequent tones. Effects on quantitative EEG (qEEG) parameters 30 min after drug administration were also evaluated. Lastly, brain samples were collected from a satellite colony of rats at a designated time point after treatment and processed to estimate the degree of NR2B receptor engagement using *ex vivo* occupancy technique.

## Materials and Methods

### Surgery

All experimental procedures were approved by the Bristol Myers Squibb Animal Care and Use Committee. Sixteen adult male Sprague-Dawley rats were anesthetized with isoflurane and implanted with epidural screw electrodes at the following coordinates (frontal; 6 mm anterior to bregma and 1 mm lateral to midline; vertex, 5.5 mm caudal to bregma and 1 mm lateral to midline; above auditory cortex, 4.8 mm caudal to bregma and 6 mm lateral to midline). Access to the electrodes was through a plastic multi-channel pedestal (Plastics One, Roanoke, VA, USA) fixed on the skull at the time of the surgery. Post-implantation, the rats were housed singly in shoe box cages and had unrestricted access to food and water. The current study used recordings from frontal and vertex leads only. Twelve of the 16 rats used were littermate controls for a neonatal treatment protocol and as such received once a day sterile saline injections on postnatal days 9–11. These and other rats were not used in any other study before electrode implantation and were thoroughly acclimated to the recording boxes and the recording tethers in the presence of brief auditory tones similar to what was used in the current study. The approximate age of the rats was 10 months at the time of the study.

### EEG recording

For EEG recordings, rats were brought to the laboratory in their home cages and placed individually in sound attenuated recording boxes equipped with a video camera, a house speaker, and a shielded light-weight cable attached to a commutator (Plastics One, Roanoke, VA, USA). Using the plastic head mounts, the cables were attached for continuous EEG recording while permitting free access to explore within the cage. For the pharmacological study, rats were first treated as per design and then placed inside the boxes. Recordings began as early as 10 or 30 min after treatment (see below).

### ERP protocols

In order to first determine if there was deviance detection, rats were subjected to the following three ERP protocols run as part of one recording session. In the first protocol, 50 ms tones of 1.0 kHz (90% probability), or 1.5 kHz (deviant; 10% probability) were presented in a random order while frames of EEG beginning 100 ms before tone onset and 250 ms after onset were sampled at a 2 kHz rate. The interstimulus interval (ISI) was fixed at 351 ms. In a second protocol, the frequencies were flipped to make 1 kHz as the infrequent stimulus (10%) and 1.5 kHz as the frequent stimulus (standard; 90%). Lastly, a third protocol was used where 10 different frequency tones including a 1.5 kHz tone (control) were presented randomly at a 10% probability each with the same ISI as before. Two difference waves were generated for each subject: by subtracting the averaged ERP to standard (1.5 kHz; 90% probability, protocol 2) from that of the deviant (1.5 kHz; 10% probability, protocol 1); and by subtracting the averaged ERP to control (1.5 kHz; 10% probability, protocol 3) from that of the deviant. To test whether the deviance was sensitive to the probability of the odd ball, protocols were tested also at 33 and 50% deviant probability. This was done on a different test session, several days after the first testing.

### Pharmacological testing

The experimental protocol used to study the effect of the NMDA antagonists on deviance detection is illustrated in Figure 3. Subjects were administered with vehicle (saline, sc) or drug (ketamine 30 mpk, ip; CP-101,606, 10 or 30 mpk sc in a cross-over design) and placed inside recording boxes. ERP protocols were run at 30 min post-dosing and 60 min post-dosing after each treatment. Additionally, a 10 min time point was included for vehicle and ketamine treatments only, to accommodate ketamine’s rapid onset of action. The 30 min test period was preceded by a 5 min free-running EEG and video recording to evaluate if the treatments had any effect on the EEG measure itself as determined by qEEG. Only ERP protocols 1 and 2 were run in this phase. Thus, there was only one difference wave generated (deviant-standard). Ketamine was sourced as the injectible drug Ketaset (Fort Dodge, IA, USA). CP-101,606 was synthesized in-house by BMS chemists. Free base mass was used for all dose calculations.

### Evoked response potentials

Single trial data were baseline corrected using the first 99 ms and low-pass filtered using a second order Butterworth IIR filter with a 30 Hz cutoff. The filtered individual trials were further visually evaluated to isolate traces that showed extreme movement-related fluctuations (typically, >150 μV but this threshold was tailored based on inspection of the individual ERPs). No more than 15% of the 1000 frames per subject were identified as unsuitable for analysis and excluded. With the remaining artifact free traces, averaged ERPs were generated for standard and deviant stimuli as described above.

### Deviance computation

Examination of the grand average ERP traces from the three protocols outlined above indicated enhanced negativity around the N1 peak (Figure 2) in the deviant ERP relative to the standard and the control ERP. Based on this, we computed area under the curve of the difference waves (deviant–standard and deviant–control) in four regions; 30–60 ms, the region encompassing the enhanced negativity and three other contiguous regions (−30 to 0, 0–30, and 60–90 ms) with zero time defined as the onset of the auditory tone. These areas were then compared to a hypothetical zero value using a two-tailed one-sample *t*-test to determine statistical significance using an adjusted *p*-value to account for multiple comparisons (<0.0125; Graphpad Prism 5.01). Consistent positive or negative deflection of the difference wave across subjects in this measure will reflect as a significant difference from zero. Moreover, we chose to use AUC rather than peak and used all available artifact free trials to construct deviant (~85), standard (~765), and control (~85). Doing this allowed us to take advantage of the statistical power inherent in a larger sample of standard ERP while avoiding a bias associated with an extreme measure such as the peak amplitude that may be distorted by unequal trials (58).

### ERP component measurement

ERP components (P1–N1–P2) were identified from averaged trace for each subject using an automated peak/trough detection feature (Signal 4.10, CED, UK) with close manual supervision. To do this, the temporal bounds for ERP components were surveyed first using the grand average of 16 subjects for each treatment condition and at each time point. These bounds served as a guide for automatic peak/trough determination for each animal in a treatment and time-specific manner. While ERP components were clearly defined under most conditions, this was not the case for a few subjects immediately after ketamine (10 min). In such cases, the highest amplitude within the time range was taken. Once the peak was determined, mean amplitude in microvolts over a 10 ms period centered on it was tabulated along with the latency. A one-way ANOVA with repeated measures followed by Bonferroni’s post-tests or Student’s *t*-test was used to determine significant effects.

To examine treatment effects, AUC_30–60 ms_ from multiple time points were compared between vehicle treatment and ketamine on one hand and vehicle and CP-101,606 (10 and 30 mpk) on the other, using two-way ANOVAs with repeated measures, using treatment as one factor and time of the ERP sampling as the second factor (Graphpad Prism 5.01). Detection of statistical significance was followed by Bonferroni post-tests between vehicle and drug treatments at designated times. A *p* < 0.05 (unless otherwise stated) was deemed to be statistically significant. To further characterize the effect of NMDA antagonists on ERPs, we looked at mean N1 amplitude of standards and deviants under all treatment conditions as outlined above.

### Free-running EEG

Thirty seconds of EEG data were chosen from each rat for Fourier analysis while reviewing the associated and time-locked video. Care was taken to choose an EEG segment that excluded slow wave activity to control for vigilance state as well as gross movement-related artifacts. The EEG segments were then Fourier transformed for absolute and relative power in the conventional frequency bands using a Hanning window to taper with 50% overlap between data blocks and an FFT block size of 1024, yielding a resolution of 0.976 Hz. Absolute and relative signal power in frequency bands (delta, 0.5–4 Hz; theta, 4–9 Hz; alpha, 9–13 Hz; beta 1, 13–19 Hz; beta 2, 20–30 Hz; gamma 1, 30–55 Hz; gamma 2, 55–100 Hz) were computed and compared between vehicle and treatments using a two-way ANOVA with treatment and frequency as the two factors. Significant treatment or frequency effects were followed-up by Bonferroni post-tests, which compared individual power bands between vehicle and drug treatments (GraphpadPrism 5.1). A *p* < 0.05 was deemed to be statistically significant.

### *Ex vivo* radioligand binding for NR2B occupancy

Rats were administered subcutaneously with CP-101,606 at 10 and 30 mg/kg [vehicle: acidified water (pH 4)]. Sixty minutes post-dose, the rats were killed and the forebrain tissues (after removal of the cerebellum and brainstem) were rapidly frozen in chilled isopentane and stored at −80°C until needed. On the day of occupancy assessment, the brain tissues were thawed and homogenized in an ice-cold homogenization buffer containing 50 mM KH2PO4, 1 mM ethylenediaminetetraacetic acid (EDTA), 0.005% Triton-X, 1:1000 dilution of Sigma protease inhibitor 3843 (pH = 7.4). The brain tissue concentration was 115 mg/ml buffer. In a 96-well plate, 23 mg of tissue (92 mg/ml) was incubated at 4°C for 5 min in 5 mM Tris–HCl (pH = 7.4) containing 5.5 nM [^3^H]RO25-6981, a highly potent and selective NR2B antagonist (59). [^3^H]RO25-6981 was synthesized by the Radiosynthesis Group in Bristol Myers Squibb. Non-specific binding was defined by inclusion of 10 μM RO25-6981. At the end of the incubation period, the reactions were stopped by filtration through FPXLR-196 filters (Brandel, Gaithersburg, MD, USA) that had been soaked in 0.5% polyethyleneimine for 1 h at 4°C. The filters were washed with ice-cold assay solution and the radioactivity was measured using a Wallac Microbeta liquid scintillation counter (Perkin Elmer Life Sciences, Boston, MA, USA). The specific binding to NR2B receptors was calculated by subtracting the value of the non-specific binding from that of the total binding in each sample. The percent occupancy was calculated as (1−specific binding in drug treated/specific binding in vehicle treated) × 100%.

### Drugs

Vehicle used in these studies was acidified sterile water for injection adjusted to a pH of 4.0 and administered subcutaneously. CP-101,606 was synthesized by BMS chemists in-house and was dissolved in pH 4.0 sterile water for injection before sc administration. Ketamine HCl was purchased in its marketed form (Ketaset, Fort Dodge, NY, USA) and was administered through the intraperitoneal route.

## Results

### ERP to auditory tones

**Figure 1:**
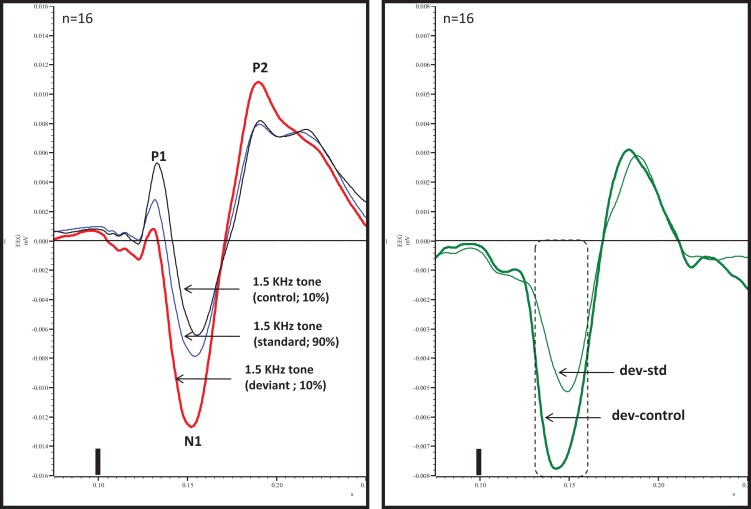
**Left panel**, overlay of standard, deviant, and control ERPs averaged from 16 rats with ERP components identified. Note prominent increased negativity under deviant condition. **Right panel**, difference waves (deviant–standard and deviant–control) indicating a prominent negativity in the region of 30–60 ms after stimulus onset. Only 30–60 ms region was significant (Bonferroni corrected *P* < 0.0125) compared to contiguous 30 ms areas (−30 to 0, 0–30, and 60–90 ms). Tone onset was defined as time 0 and is marked by a vertical bar in this and all other figures that show ERP traces.

**Figure 2:**
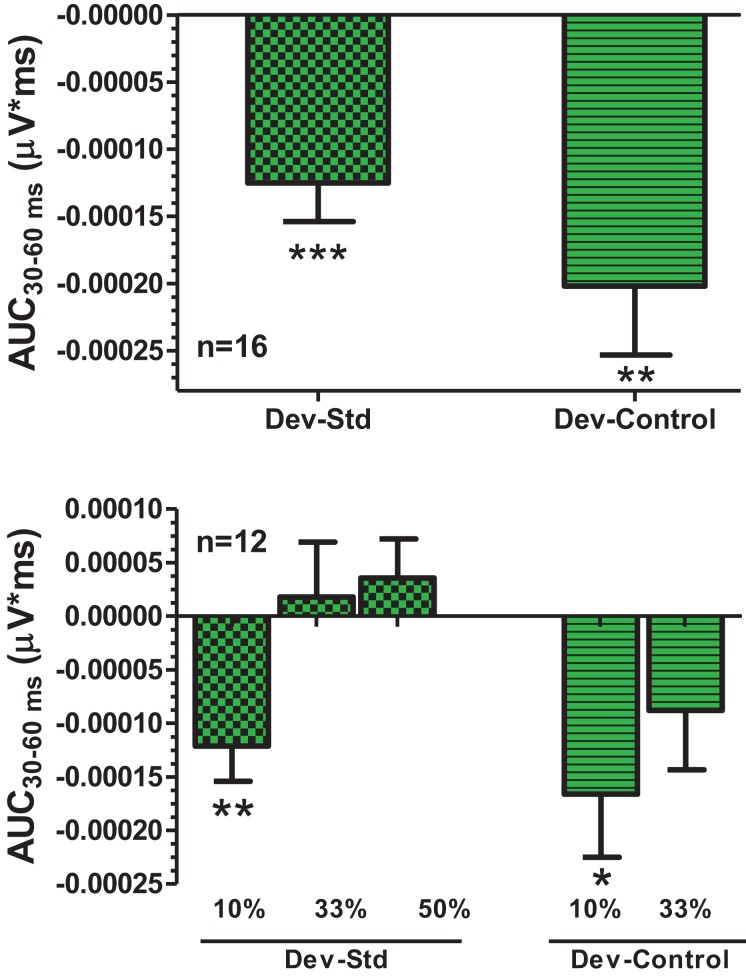
**Top panel**, AUC_30–60 ms_ areas from difference waves show robust significance against a hypothetical zero value (Bonferroni corrected *P* < 0.0125). **Bottom panel**, AUC_30–60 ms_ was robustly sensitive to deviant probability.

An overlay of grand averaged ERPs from 16 subjects in response to a 1.5 kHz tone delivered as a standard, control, or deviant are shown in Figure 1 (left panel). The grand averaged ERPs showed a prominent positive, negative, and positive components identified as P1, N1, and P2, respectively. The grand averaged difference waves were obtained by subtracting the standard or the control ERP from that of the deviant ERP and displayed in Figure 1 (right panel). The two difference waves showed a similar pattern of a distinct negative peak around 50 ms after stimulus onset. Consequently, areas under the curve were computed for the visually identified region of negativity 30–60 ms post-stimulus onset for both difference waves. These measures showed a robust deviance from zero (Figure 2, top panel) using a corrected *p*-value (0.0125) while no other contiguous bands of area, i.e., −30 to 0 ms; 0–30 ms; 60–90 ms showed any significant deviance, positive or negative, from zero (data not shown). Significantly, there was no difference between the 30 and 60 ms areas generated by the two difference waves; that is, deviant–standard and deviant–control generated similar negativity. Increasing odd-ball probability from 10 to 33% or higher resulted in the elimination of the deviance from both the difference waves (Figure 2, bottom panel). Since the peak negativity in the difference waves was coincidental with the N1 response, we hypothesized that it is being driven by an increased N1 in the deviant waveform. We, therefore, tested the correlation between the AUC_30–60 ms_ of the respective difference waves with that of N1 amplitude difference between standard or control and deviant and found robust and highly significant correlations (*r*^2^ ≥ 0.89; *p* < 0.0001 for both sets of data). The mean amplitude and latency of each of the three ERP components are summarized in Table 1. One-way ANOVA analysis showed robust effects on P1 (and N1) amplitudes depending on whether the ERP components are derived from a deviant or a standard/control ERP. Subsequent Bonferroni *post hoc* tests established a significant reduction in P1 (and an enhancement in N1) of the deviant waveform, compared to the standard and the control. No such difference was noted for the P2 response. Lastly, component latencies were unaffected across the three conditions (Table 1).

**Figure 3:**
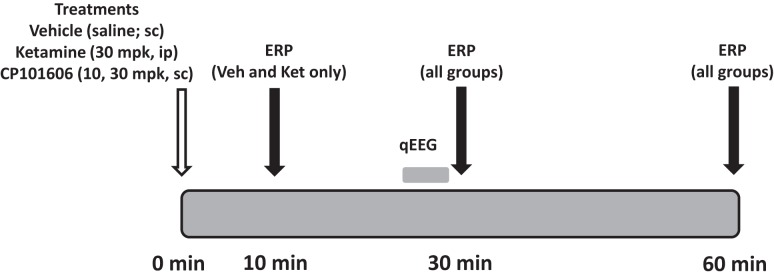
**Schematic indicates protocol timeline**. Treatments were made in a randomized cross-over design on 16 subjects at time zero. EEG was collected at three time points post-dosing.

**Table 1:** **ERP component latency and amplitude under three conditions of evocation**.

ERP	P1		N1		P2	
	Latency (ms) (range)	Mean amplitude (μV)	Latency (ms) (range)	Mean amplitude (μV)	Latency (ms) (range)	Mean amplitude (μV)
Standard	28.70 ± 1.66	3.60 ± 1.07*	52.87 ± 2.61	−9.77 ± 1.44*	94.16 ± 2.62	8.39 ± 1.47
Control	32.45 ± 0.97	4.81 ± 1.40**	54.30 ± 2.03	−9.08 ± 1.64**	90.02 ± 4.78	9.23 ± 1.70
Deviant	28.43 ± 2.04	1.12 ± 1.30	52.35 ± 1.80	−13.87 ± 1.86	91.00 ± 2.93	11.07 ± 1.42
One-way ANOVA		*F*(2,15) = 7.46; *P* = 0.0023		*F*(2,15) = 6.02; *P* = 0.0063		

*/**Indicate significant difference from corresponding deviant (*p* < 0.05/0.01; one-way ANOVA followed by Bonferroni post-tests).

### Effect of NMDA antagonists on deviance detection

The effects of non-selective and selective NMDA blockers on deviance detection were tested in a cross-over study. These studies were initiated in the same rats, which showed deviance detection as above, after a gap of several days. However, to accommodate testing at multiple time points, we simplified the ERP protocol to collect only standard and deviant ERPs. Thus, a control condition was not used in this part of the study.

### Ketamine effects on deviance detection

**Figure 4:**
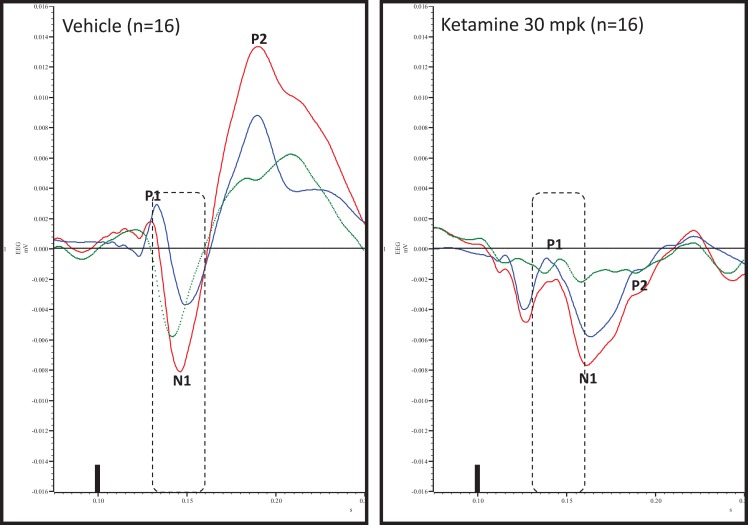
**Overlay of standard (blue), deviant (red), and difference wave (deviant–standard; green) ERPs approximately 10 min after vehicle or ketamine treatment is shown**. Notice a well-defined negativity in the difference wave 30–60 ms (dashed box) post-stimulus onset after vehicle treatment only. Also notice a visible delay in N1 after ketamine treatment.

**Figure 5:**
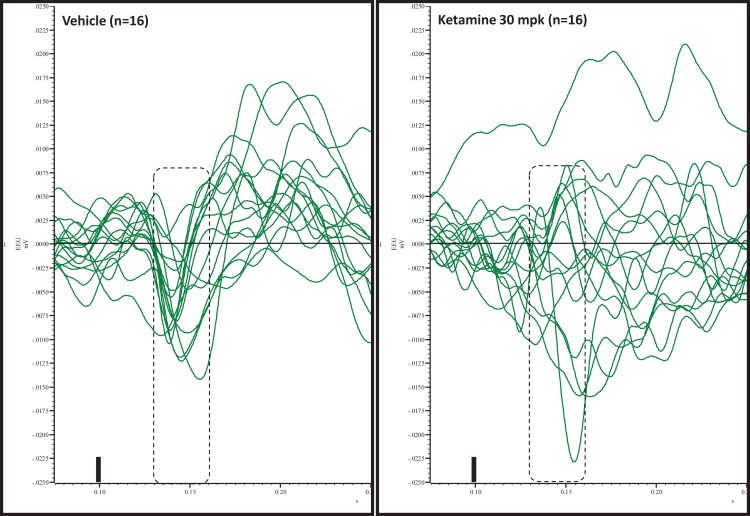
**Overlay of difference waves after vehicle or ketamine approximately 10 min after treatment**. A consistent pattern of negativity is apparent after vehicle but not after ketamine.

**Figure 6:**
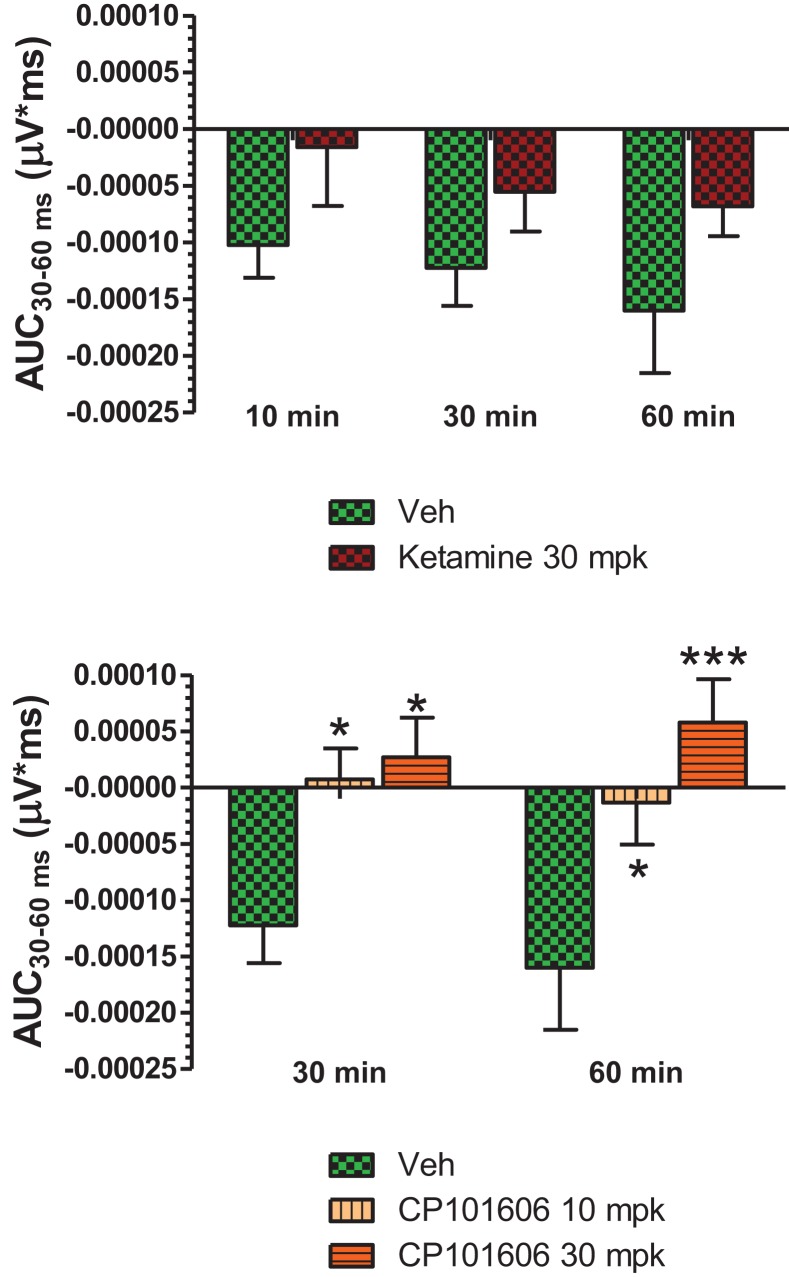
**Top panel**, AUC_30–60 ms_ of difference waves contrasted after vehicle or ketamine (10 mg/kg, ip) treatment at three time points. Two-way ANOVA with repeated measures revealed a strong overall treatment effect [*F*(1) = 8.44; *P* = 0.0068]. **Bottom panel**, AUC_30–60 ms_ values contrasted after vehicle and CP-101,606 (10 or 30 mg/kg, sc) treatment. Two-way ANOVA with repeated measures showed a strong overall treatment effect [*F*(2) = 11.86; *p* < 0.0001]. */***Indicates significant difference (*P* < 0.05/*P* < 0.0001) between vehicle and respective CP-101,606 treatment.

**Figure 7:**
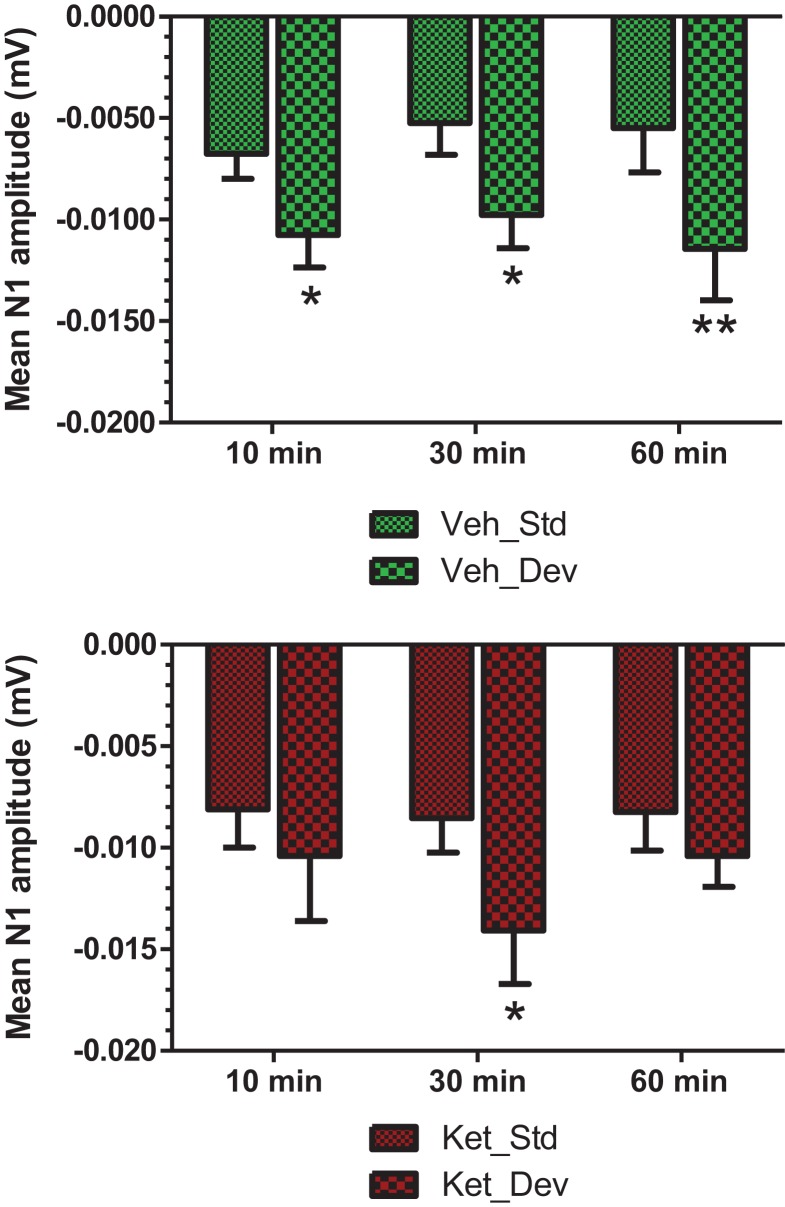
**Standard and deviant N1 amplitudes compared are shown**. Top panel summarizes post-vehicle responses across three time points. Two-way ANOVA with repeated measures showed a robust effect of condition (standard or deviant) on N1 response [*F*(1) = 27.85; *P* < 0.0001]. Bottom panel shows post-ketamine data. A significant condition effect was noted [*F*(1) = 6.82; *P* = 0.012]. */**Indicate significant treatment effect (*P* < 0.05/0.01 respectively; Bonferroni post-tests).

**Figure 8:**
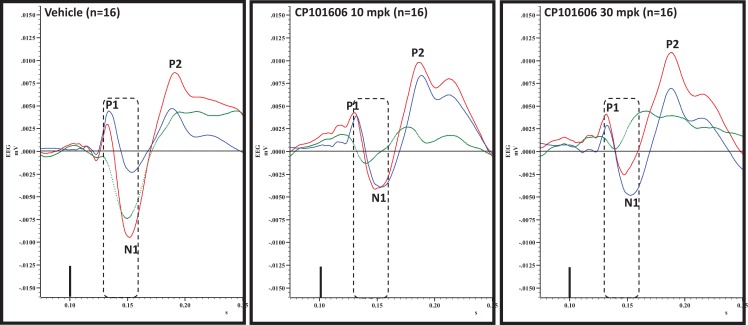
**Overlay of standard (blue), deviant (red) and difference wave (deviant-standard; green) ERPs approximately 60 min after vehicle or CP-101606 (10 or 30 mg/kg) treatment is shown**. Notice a well-defined negativity in the difference wave 30–60 ms post-stimulus onset (dashed box) after vehicle treatment only.

Grand averaged ERPs to standard, deviant, and the difference wave are shown overlaid for the 10 min time point after vehicle or ketamine treatment (Figure 4) and indicate a sharply defined difference wave after vehicle only. Butterfly plots of individual difference waves 10 min after treatment confirm this difference (Figure 5) and indicate a lack of temporally consistent pattern of negativity across 16 subjects following ketamine dosing. Deviance computed as AUC_30–60 ms_ across three time points is shown in Figure 6 (top panel). A robustly significant treatment effect [*F*(1) = 8.44; *P* = 0.0068] but no significant time or treatment × time interaction was noted. While mean AUC_30–60 ms_ measure was consistently lower under ketamine vs. vehicle, Bonferroni post-tests found the difference at individual time points to be not significant (*P* > 0.05). Since N1 components were visibly delayed under ketamine treatment (Figure 4), it is possible that we were underestimating the deviance by using the 30–60 ms time window for the AUC determination. To address this, we measured mean N1 amplitude of standard and deviant under both treatments for each subject as outlined in the Section “Materials and Methods”. These data are summarized in Figure 7. As expected, under vehicle condition (Figure 7, top panel) there was a robust treatment effect [*F*(1) = 27.85; *P* < 0.0001] but no treatment or treatment × time interaction (*P* > 0.05). Moreover, Bonferroni post-tests showed significant difference between standard and deviant at all three time points (Figure 7, top panel). Whereas under ketamine treatment, while there was a significant treatment effect [*F*(1) = 6.82; *P* = 0.012], only the 30 min time point showed a significant difference (Figure 8, bottom panel). Moreover, there was a small but significant treatment effect in the form of an overall augmentation in standard N1 amplitude under ketamine condition when compared to N1 under vehicle [*F*(1) = 5.55; *P* = 0.022] (Table 2). There were, however, no time or time × treatment effects noted (*P* > 0.05) (Table 2). No significant effects were noted on the deviant N1 ERP amplitude. However, robust treatment effects were noted on standard as well as deviant N1 latencies (Table 2) in the form of significant delays. Robust treatment effects were noted on the P2 component as well; ketamine suppressed and delayed P2 component of standard ERP (Table 3). Deviant P2 component was suppressed as well but the delay in latency was not significant (*P* > 0.05) (Table 3). Moreover, a significant treatment effect only was noted on the P1 component of standard ERP where ketamine suppressed P1 amplitude [*F*(1) = 5.49; *P* = 0.023; data not shown]. Bonferroni post-tests showed no significance between vehicle and ketamine treatments on P1 at any of the time points. P1 latency was unaffected (data not shown). No significant P1 effects were noted on the deviant ERP (data not shown). Thus ketamine treatment profoundly altered the amplitude and latency of multiple ERP components in a time-sensitive fashion.

**Table 2:** **N1 component latency and amplitude tabulated as standard and deviant across vehicle and ketamine treatments**.

N1 only	Vehicle		Ketamine 30 mpk	
ERP	Latency (ms)	Mean amplitude (μV)	Latency (ms)	Mean amplitude (μV)
Standard_10 min_	50.2 ± 2.0	6.7 ± 1.2	67.5 ± 2.9***	8.1 ± 1.8
Standard_30 min_	54.3 ± 2.2	5.2 ± 1.5	58.3 ± 2.3	8.5 ± 1.7
Standard_60 min_	52.4 ± 2.4	5.5 ± 2.1	53.4 ± 2.9	8.2 ± 1.9
Two-way ANOVA			Treatment; *F*(1) = 15.67; *P* = 0.0003	Treatment; *F*(1) = 5.55; *P* = 0.02;
			Treat × Time; *F*(2) = 7.03; *P* = 0.0022	Treat × Time, ns
			Time, ns	Time, ns
Deviant_10 min_	46.2 ± 1.7	10.7 ± 1.6	65.4 ± 2.5***	10.4 ± 3.2
Deviant_30 min_	50.2 ± 1.8	9.7 ± 1.6	57.3 ± 2.4	14.0 ± 2.6
Deviant_60 min_	50.8 ± 1.7	11.4 ± 2.5	49.5 ± 3.3	10.4 ± 1.5
Two-way ANOVA			Treatment; *F*(1) = 21.8; *P* < 0.0001	Treatment, ns
			Treat × Time; *F*(2) = 11.2; *P* = 0.0001	Treat × Time, ns
			Time, ns	Time, ns

***Indicate *p* < 0.001 (Bonferroni post-tests) when compared to corresponding value under vehicle condition. Note a robust latency delay and an augmentation of amplitude (standard only) under ketamine.

**Table 3:** **N2 component latency and amplitude tabulated as standard and deviant across vehicle and ketamine treatments**.

P2 only	Vehicle		Ketamine 30 mpk	
ERP	Latency (ms)	Mean Amplitude (μV)	Latency (ms)	Mean Amplitude (μV)
Standard_10 min_	85.6 ± 2.4	9.2 ± 1.4	103.5 ± 6.5**	2.9 ± 1.5***
Standard_30 min_	89.8 ± 2.2	5.7 ± 1.5	91.4 ± 2.4	3.1 ± 1.1**
Standard_60 min_	85.6 ± 4.2	5.1 ± 1.5	87.4 ± 3.0	5.7 ± 1.4
Two-way ANOVA			Treatment; *F*(1) = 5.48; *P* = 0.023	Treatment; *F*(1) = 11.79; *P* = 0.003
			Treat × Time; *F*(2) = 3.2; *P* = 0.0501	Treat × Time; *F*(2) = 6.00; *P* = 0.0049
			Time, ns	Time, ns
Deviant_10 min_	88.0 ± 2.4	12.7 ± 1.7	95.1 ± 6.7	3.9 ± 1.9***
Deviant_30 min_	87.8 ± 2.0	9.6 ± 2.0	88.5 ± 2.8	2.3 ± 1.4**
Deviant_60 min_	89.0 ± 2.6	9.6 ± 1.8	88.4 ± 2.8	8.5 ± 1.8
Two-way ANOVA			Treatment, ns	Treatment; *F*(1) = 20.14; *P* < 0.0001
			Treat × Time, ns	Treat × Time; *F*(2) = 3.43; *P* = 0.041
			Time, ns	Time, ns

**/***Indicate *p* < 0.01/0.001 (Bonferroni post-tests) when compared to corresponding value under vehicle condition. Note a robust latency delay (standard only) and a suppression of amplitude under ketamine that wanes over time.

### Effect of selective NR2B antagonist CP-101,606 on deviance detection

**Figure 9:**
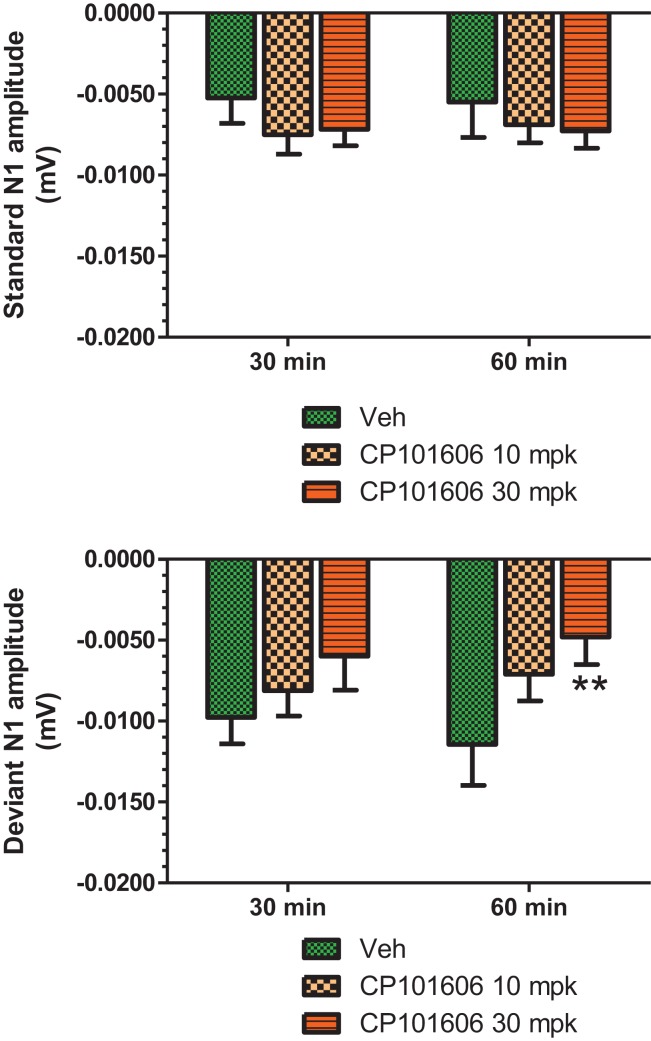
**Standard and deviant N1 ERP amplitudes after vehicle or CP-101,606 (10 or 30 mg/kg) treatment are shown**. Top panel shows standard N1 amplitudes and bottom panel shows deviant N1 amplitudes. Notice that only deviants after CP101,606 show apparent dose-dependent reduction in N1 amplitude. **Indicates *P* < 0.01 (BonferroniŠs post-tests).

An overlay of the standard, deviant, and difference ERPs 60 min after vehicle or CP-101,606 treatment are shown in Figure 8. Whereas a robust negativity of the difference wave is apparent in the 30–60 ms period after stimulus onset after vehicle, there is little negativity under CP-101,606 treatment. To confirm, AUC_30–60 min_ across two time points (30 and 60 min after dosing) were compared between vehicle and CP-101,606, using a two-way ANOVA with repeated measures. A highly significant treatment effect [*F*(2) = 11.86; *p* < 0.0001] but no significant time or treatment × time interaction effects (*P* > 0.05) were noted. Further, Bonferroni tests revealed significant difference between vehicle and CP-101,606 (10 and 30 mpk; *p* < 0.05 or greater) at every time point. These results are summarized in Figure 6 (bottom panel). Although difference waves were clearly affected by the NR2B antagonist, to document if this is driven by the drug response to standard or deviant or both, we looked at mean N1 amplitude of standard and deviant ERPs under vehicle and CP-101,606 (10 and 30 mpk) treatments using a two-way ANOVA. Standard ERPs did not differ in amplitude or latency between treatments (Figure 9, top panel). On the other hand, a robust treatment effect in the form of an amplitude reduction was apparent in deviant N1 [*F*(2) = 6.01; *P* = 0.0042]. Bonferroni post-tests showed a robust suppression of the deviant N1 treated with CP-101,606 (60 min; 30 mpk only) compared to vehicle response (Figure 9, bottom panel). No treatment effects on latency were noted for either the standard or the deviant ERPs (data not shown). To test whether any significant deviance detection under CP-101,606 treatment remained, we directly compared N1 of the deviant and standard ERPs at the two time points at both doses using paired *t*-tests. No significant differences were found (data not shown). Interestingly, at the 30 mpk dose only, there was a trend level significance for treatment [*F*(1) = 2.28; *P* = 0.1008], indicating a trend toward a smaller deviant relative to the standard. The two remaining ERP components of the vertex potentials, namely P1 and P2 were also scrutinized. No treatment effect or time effect or their interaction was significant across groups, suggesting that these components were unaffected by CP-101,606 (data not shown).

### Effect of non-selective and selective blockers of NMDA transmission on quantitative EEG measures

**Figure 10:**
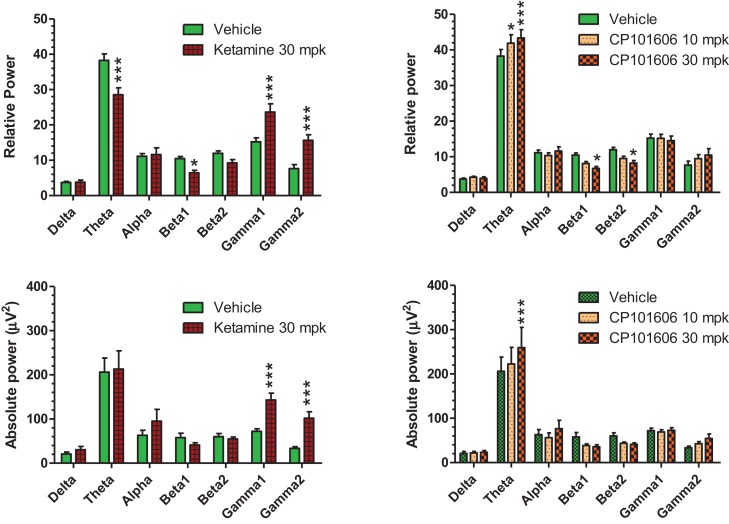
**Summary graphs of quantitative EEG parameters analyzed approximately 30 min after vehicle or ketamine (30 mg/kg, ip), or CP-101,606 (10 or 30 mg/kg, sc) treatments**. */***Indicate *P* < 0.05/*P* < 0.0001, respectively (Bonferroni post-tests).

Careful EEG review along with synchronized video feed found little evidence for sleep under the recording conditions. Moreover, a dominant theta oscillatory activity, indicative of a wakeful state, was apparent in all subjects (Figure 10), irrespective of the treatment. As expected, ketamine produced robust increases in absolute and relative gamma power (Figure 10, left panels). A robust reduction in relative theta power and a small but significant reduction in beta 1 relative power were also noted under ketamine. In contrast, there was an overall increase in theta power under CP-101,606 treatment and a small but significant reduction in beta 1 and 2 relative powers at the 30 mg/kg dose only (Figure 10, top right panel). No other bands were significantly affected with CP-101,606 treatment.

### *Ex vivo* NR2B occupancy

A small group of rats were dosed with CP-101,606 (10 and 30 mpk sc; *n* = 4/dose) and brain samples were collected 60 min post-dosing for *ex vivo* occupancy. A robust occupancy of the NR2B receptor at both doses was noted (79 ± 4% at 10 mpk sc and 87 ± 8% at 30 mpk sc compared to 0 ± 5% under vehicle condition). These results are in agreement with rodent *ex vivo* occupancy of CP-101,606 reported previously (60, 61). Ketamine occupancy was not studied.

## Discussion

Using two different but complementary approaches to register deviance, we found reproducible deviance detection in the vertex ERPs of conscious rats, centered on the N1 response. This effect was reproducible across multiple sessions and was highly sensitive to deviant probability. Deviance as defined by deviant–standard ERP was attenuated by the non-selective NMDA channel blocker ketamine, validating our approach. On the other hand, the complete disruption of deviance detection by a selective NR2B blocker suggests that these subunits play a vital and hitherto unexplored role in automatic deviance detection.

Recent rodent literature on MMN has focused on establishing whether there is deviance detection in the auditory cortex of rats. In contrast to some reports in the past that failed to find an MMN-like response in the primary auditory cortex of anesthetized (19) or awake rats (20), many recent rat studies show deviance detection either as an increase in negativity (22, 62, 63) or an increased positivity (63), in response to the occasional odd ball by focusing on epidural ERPs. Moreover, these responses are sensitive to the probability of deviance and are demonstrable using the rigorous “many standards” condition first proposed by Jacobsen and Schroeger (64). The qualitative difference in findings between extracellular single-unit recordings and local field potentials on one hand and ERPs on the other appears to be critical (7). Whereas the former samples from a limited and highly localized group of neurons, epidural ERPs reflect activity from multiple brain regions, including subcortical sources as in the case of midline vertex potentials. Of course the limitation of an epidural ERP measure is the inability to unequivocally determine the primary source or generator of the deviance signal, without extensive further investigation. In the current studies, we have focused on midline vertex ERPs that are not solely dependent on input from the primary auditory cortex (26), a key source for auditory MMN. Instead, midline ERP responses are more symmetric across the hemispheres and may reflect activity from midline subcortical structures that are not a part of the primary lemniscal pathway (24, 25, 65) as well as structures such as the hippocampus and the anterior cingulate (26). Since deviance detection may be a fundamental ability required for species survival, it is not surprising that there are parallel pathways to support this vital function.

We saw robust separation between deviant and standard or control beginning about 30 ms post-stimulus onset that peaked within the following 30 ms. It is possible that the relatively brief tone duration of 50 ms may have punctuated the negativity as a robust “off” response at the end of the auditory stimulus was always prominent culminating in the P2 response. Indeed, it was recently noted that by prolonging the stimulus duration, the duration of negativity could be improved (62).

Overall, the only consistent increased negativity we saw hovered around the N1 response and clearly distinguished the larger deviant response from standard. One criticism about this comparison is that since the standard is repeated more frequently than the deviant, there is a greater neuronal habituation to the standard stimulus relative to the infrequent deviant and that this does not represent a true memory trace that is presumed to underlie MMN. Nevertheless, such a habituation is evidence of a pre-attentive ability to discriminate a stimulus by means of its context (repetitive or infrequent). To discount habituation as the only viable mechanism of deviance detection, however, Jacobsen and Schroeger designed a control protocol in which multiple stimuli including the deviant tone are presented randomly at a probability identical to that of the deviant (64). Since the presentation rate of the stimulus is same as the deviant, it is expected to have little additional inhibition. Moreover, because it is presented randomly as part of many other tones, no necessary context for novelty exists unlike a typical odd ball, which is presented rarely amidst a stream of identical standards. Significant difference between a stimulus delivered as part of a control protocol vs. the same stimulus delivered as an odd ball is argued to represent true deviance. We saw robust negativity under standard as well as control conditions suggesting genuine deviance detection in the vertex potentials. Moreover, the difference wave generated by either condition was robustly sensitive to deviant probability with both 33 and 50% evoking no significant deviance, suggesting that low deviant probability is critical for establishing a context of regularity. Within the same group of rats used in the current studies, the deviance identified as negativity between 30 and 60 ms after stimulus onset was consistent within as well as across sessions, allowing us to study the effect of the NMDA antagonists in a balanced cross-over design that lasted a few weeks.

Often MMN has been defined operationally as a late negativity that appears subsequent to the N1 response. Yet in practice, it is common for the deviance to appear even before the N1 has peaked and therefore, showing a clear overlap in the deviant N1 recovery trajectory and that of the MMN. This is particularly the case when there is a robust spectral separation between standard and deviant tones as in the current study (66). A close examination of frequency MMN papers that include standard, deviant, and MMN traces supports this notion of overlap (67–69). Indeed, May and Tinnen (70) as well as others (71) have argued that MMN is not distinct from an N1 response modulated by stimulus contingency. We found a strong correlation between AUC_30–60 ms_ and the amplitude difference between deviant and standard ERPs on one hand and deviant and control ERPs on the other, establishing that deviance in our studies was being driven by a heightened N1 response to novel stimuli. Additionally, we saw no consistent protracted negativity in the difference wave going beyond the N1 time frame. This was unexpected since the midline vertex potentials in Guinea pigs showed robust negativity that not only had an early onset similar to our finding but also continued throughout the subsequent ERP trace (24, 25). However, unlike in our data, they did not see an “off” response that culminated in a P2 wave. In future studies, it would be interesting to see if the negativity can be prolonged by prolonging the tone duration as has been shown recently (62).

In addition to the N1 component being driven by novelty, we also saw evidence for P1 component in the deviant ERP being substantially smaller than both standard and control ERPs, suggesting that even as early as 25 ms after stimulus onset, a deviance was being registered. Although we have not seen literature evidence for such early deviance in preclinical ERP recordings, stimulus-specific adaptation, a putative mechanism for deviance detection, has been reported as early as 20 ms from stimulus onset in both the inferior colliculus and the dorsal portion of the medial geniculate, structures that mediate the non-lemniscal processing of auditory signals (72, 73). Thus, the observed early changes in vertex recordings may reflect activity from these subcortical structures. While deviance detection in components earlier than N1 is not generally reported or discussed typically in the clinical literature on MMN, a recent report has found evidence for deviance detection as early as 40 ms in human scalp EEG (74). It has been argued that N1–MMN–N2b may represent a continuum of deviance detection (1). Support for early auditory detection of deviance including some that precedes MMN has been reviewed recently (14, 75).

Attenuation of MMN by NMDA antagonists has been an important pharmacological validation ever since it was first demonstrated in monkeys with PCP (28), and subsequently replicated in multiple species including human, primate, and rodent (5, 30, 31, 33, 56, 76, 77). Moreover, ketamine, a fast acting, non-selective NMDA channel blocker with rapid pharmacokinetics has been frequently studied for its effects on MMN in healthy humans. Although a few studies have failed to demonstrate significant modulation (55, 78, 79), a majority reported diminution of MMN. In studies where ketamine diminished deviance response, the basis is often unclear since the difference wave is a virtual construct materialized by subtracting the standard from the deviant and frequently standard and deviant ERPs are not shown.

Given the temporal overlap between standard, deviant, and the difference wave in our study, we suspected that the N1 component could be contributing to the reduced deviance following ketamine administration. While ketamine treatment clearly delayed the appearance of ERP components like N1, the overall ERP morphology was preserved, allowing component measurements. A significant disinhibition of the standard N1 across three time points was noted under ketamine (Table 2). Since a standard ERP is presented many more times than a deviant, it is under a greater inhibition (i.e., stimulus-specific adaptation). It is this inhibition that ketamine appears to relieve somewhat. This effect clearly contributed to the diminished significance between the deviant and standard ERPs under ketamine. This observation supports the contention that stimulus-specific adaptation is a key mechanism underlying deviance detection and non-selective NMDA blockers interfere with this process. A similar observation was previously made by Ehrlichman and colleagues using mouse hippocampal field recordings (56). That ketamine has disinhibitory effects on ERPs is not unprecedented as at least two clinical studies documented disinhibitory effect of ketamine on N1 amplitude (33, 55) while using an odd-ball paradigm. Other reports have described an augmentation of sensory or somatosensory ERPs under ketamine (80, 81), perhaps a result of cortical glutamate efflux and activation of non-NMDA mediated excitatory transmission through AMPA receptors (82). However, others have found no effect on N1 amplitude in an odd-ball protocol (76). We speculate that these differences can be attributed to variable dose and testing regimens that in turn affect the degree of NMDA channel blockade obtained in these studies. A careful temporal characterization of ketamine dose–response on ERPs generated by an odd-ball protocol could reveal the excitatory and inhibitory effects of ketamine on ERPs and reconcile these differences.

Apart from its amplitude effects, ketamine robustly suppressed and delayed the latency of ERPs, especially at the first time point (~10 min post-injection), suggesting a relative slowing of signal processing and delay in peak synchrony of the neural oscillators generating the ERP response. Ketamine-induced slowing of ERPs has been reported both in clinical (30, 83) as well as in experimental subjects previously (56, 84, 85). Apart from its effects on N1 amplitude and latency, Ketamine impacted other ERP components as well. Generally, both P1 and P2 components tended to be suppressed relative to vehicle at the 10 and 30 min time points and recovered by the 60 min time point. Compared to P1, effects on P2 were more robust. Lastly, ketamine’s effects on qEEG have been well characterized previously and the current results are in line with these findings (86, 87). In addition, the dominant power of theta band is indicative of the strong hippocampal contribution to the vertex recorded EEG.

Perhaps, the most significant finding of our report was the complete elimination of deviance after treatment with CP-101,606, a highly selective antagonist of NR2B receptors (88, 89). In contrast to ketamine, no significant disinhibition of the standard N1 response was noted. Instead, there was a dose-dependent and robust suppression of the deviant N1 response. This suggests that NR2B receptors may be mediating true context-based deviance detection rather than stimulus-specific adaptation alone. For example, if NR2B receptors affected adaptation, one would expect a clear increase in standard ERPs and consequently, no significant difference between the less suppressed deviant and the disinhibited standard. Instead, we saw the opposite; i.e., a significant and dose-dependent suppression of the deviant and no significant disinhibition of the standard ERP. Since a shift in attention involving frontal cortical regions is believed to be responsible for enhanced deviant negativity (44, 46, 48, 90), we can speculate that the NR2B mechanism may be vital for mediating this function and that selective NR2B antagonists interfere with this process. Moreover, it has been recently argued that whereas sensory cortices participate in deviance detection by means of stimulus-specific adaptation (where neighboring cortical columns are suppressed from responding to repeated stimuli), connectivity from frontal regions such as the medial prefrontal cortex to the temporal cortex modulates the gain of this process in an NMDA-dependent mechanism (31). Consistent with this hypothesis, patients with localized lesions within the medial prefrontal cortex have poor deviance detection (42, 43). Moreover, schizophrenia patients as a group have highly reproducible and stable deficits in MMN even as they consistently show deficits in cognitive tasks that are driven by this region such as working memory and executive function (45, 91–93). Prefrontal cortical neurotransmission is believed to be especially sensitive to NMDA transmission (94, 95). Recently, there have been several reports that show that NR2B-based neurotransmission is critical for the optimal function of this region (39, 41, 96, 97). We speculate that disruption of NR2B neurotransmission in prefrontal cortex may contribute to the loss of deviance detection in NR2B-treated rats. Interestingly, a recent report showed molecular evidence for potential aberrant trafficking of NR2B receptors, but not NR2A, within cortical layers of schizophrenia patients (98). The differential effects of CP-101,606 and ketamine on qEEG parameters further highlight how individual subunit modulation can alter the field response in a way that is not reflected by non-selective channel blockers.

To conclude, robust deviance detection to pitch changes were noted in vertex potentials of awake and freely behaving rats. The deviance detection was diminished by pretreatment with the non-selective NMDA channel blocker ketamine which disinhibited and enlarged standard ERP response. On the other hand, the NR2B selective antagonist CP-101,606 completely abolished deviance detection by selectively inhibiting the deviant ERPs. This is the first demonstration that reducing function of NR2B receptors disrupts the pre-attentive auditory deviance detection mechanism in rats.

## Conflict of Interest Statement

The authors declare that the research was conducted in the absence of any commercial or financial relationships that could be construed as a potential conflict of interest.
